# Stable oxygen isotope and flux partitioning demonstrates understory of an oak savanna contributes up to half of ecosystem carbon and water exchange

**DOI:** 10.3389/fpls.2014.00530

**Published:** 2014-10-07

**Authors:** Maren Dubbert, Arndt Piayda, Matthias Cuntz, Alexandra C. Correia, Filipe Costa e Silva, Joao S. Pereira, Christiane Werner

**Affiliations:** ^1^Agroecosystem Research, University of Bayreuth, BayCEERBayreuth, Germany; ^2^Computational Hydrosystems, Helmholtz Center for Environmental Research (UFZ)Leipzig, Germany; ^3^Instituto Superior de Agronomia, University of LisbonLisbon, Portugal

**Keywords:** partitioning, stable oxygen isotopes, evapotranspiration, savanna, dry-land ecosystems, net ecosystem CO_2_ exchange, water-use efficiency, soil infiltration

## Abstract

Semi-arid ecosystems contribute about 40% to global net primary production (*GPP*) even though water is a major factor limiting carbon uptake. Evapotranspiration (*ET)* accounts for up to 95% of the water loss and in addition, vegetation can also mitigate drought effects by altering soil water distribution. Hence, partitioning of carbon and water fluxes between the soil and vegetation components is crucial to gain mechanistic understanding of vegetation effects on carbon and water cycling. However, the possible impact of herbaceous vegetation in savanna type ecosystems is often overlooked. Therefore, we aimed at quantifying understory vegetation effects on the water balance and productivity of a Mediterranean oak savanna. *ET* and net ecosystem CO_2_ exchange (*NEE*) were partitioned based on flux and stable oxygen isotope measurements and also rain infiltration was estimated. The understory vegetation contributed importantly to total ecosystem *ET* and *GPP* with a maximum of 43 and 51%, respectively. It reached water-use efficiencies (*WUE;* ratio of carbon gain by water loss) similar to cork-oak trees. The understory vegetation inhibited soil evaporation (*E*) and, although *E* was large during wet periods, it did not diminish *WUE* during water-limited times. The understory strongly increased soil water infiltration, specifically following major rain events. At the same time, the understory itself was vulnerable to drought, which led to an earlier senescence of the understory growing under trees as compared to open areas, due to competition for water. Thus, beneficial understory effects are dominant and contribute to the resilience of this ecosystem. At the same time the vulnerability of the understory to drought suggests that future climate change scenarios for the Mediterranean basin threaten understory development. This in turn will very likely diminish beneficial understory effects like infiltration and ground water recharge and therefore ecosystem resilience to drought.

## Introduction

Semi-arid ecosystems contribute about 40% to global net primary productivity (Wang et al., 2012) and in these ecosystems water and carbon dioxide cycles are tightly coupled via ecosystem water use efficiency (David et al., 2004; Pereira et al., 2006). Global climate change is expected to intensify drought and alter precipitation patterns in dry-land regions (IPCC, 2007). Moreover, evapotranspiration (*ET*) accounts for up to 95% of the water loss from the ecosystem (Huxman et al., 2005). *ET* has two distinct components: plant transpiration (*T*) and unproductive loss of water during soil evaporation (*E*). Due to their open bi-layered structure, savanna-type ecosystems are particularly suitable to study the effect of water scarcity and the coupling between hydrological and biogeochemical processes of different plant layers (woody vs. herbaceous species) and the soil. They cover large areas world-wide and in Europe they are the predominant land cover type on the southern Iberian Peninsula, covering about 1.5 Mio ha (Bugalho et al., 2011). They consist of a sparse tree cover (e.g., cork-oak, *Quercus suber L*., and holm oak, *Q. ilex*) and an herbaceous understory layer. They are exploited as often low-impact agro-forestry ecosystems with high biodiversity, specifically of the herbaceous layer, and considered a habitat of high conservation value (Moreno et al., 2005; Perez-Ramos et al., 2008). Hence, their sustainability is vitally important for both agronomical and biodiversity aspects, but is currently being threatened by unbalanced management practices (Bugalho et al., 2011). Moreover, while trees have access to deeper soil layers and/or groundwater, shallow rooted herbaceous plants are vulnerable to drought, and die back at the onset of summer drought (Paço et al., 2009). Still, the herbaceous understory vegetation has a strong impact on ecosystem productivity: it can make up to more than 50% of total gross primary productivity (*GPP*) during spring (Unger et al., 2009, 2010).

While the impact of herbaceous plants and soil on carbon cycling in oak savannas is relatively well-characterized, less is known concerning their role in the ecosystem water cycle. In general, introducing dense herbaceous layers to maximize the productive and minimize the unproductive water loss by reducing open soil patches (Wang et al., 2010; Raz-Yaseef et al., 2012) has been considered a major goal in dry-lands (Wang and D'Odorico, 2008). However, the presence of (herbaceous) vegetation has various other impacts on soil water relations than sheer reduction of soil evaporation. Rainfall might be intercepted while at the same time hydraulic redistribution might be altered depending on rooting depths and structure (Tromble, 1988; Dawson, 1993; Schwinning and Ehleringer, 2001; Devitt and Smith, 2002; Bhark and Small, 2003; Huxman et al., 2005; Scott et al., 2014). Moreover, transpiration of active vegetation can have a huge impact on ecosystem water losses which are modulated by water availability, plant functional type, and stomatal regulation, as well as leaf area index (*LAI*). Paço et al. (2009) gave first insights that at least in times of high water availability (October-May/June) understory evapotranspiration can be equal to and sometimes exceeds tree transpiration. Soil evaporation and herbaceous transpiration, however, have seldom been analyzed separately in savanna ecosystems so far. Thus, the functional understanding of soil evaporation dynamics and vegetation-soil feedbacks within the water cycle remain a major challenge in semi-arid regions.

Consequently, in dry-land ecosystems partitioning *ET* and analyzing vegetation effects on soil water distribution is not only important to better understand the ecosystem water budget (Haverd et al., 2011; Raz-Yaseef et al., 2012) but also for predictions of carbon cycling, i.e., ecosystem productivity (Scott et al., 2006; IPCC, 2007; Yepez et al., 2007). Oxygen isotope signatures (δ*^18^O*) have been used to partition ecosystem *ET* because of the distinct isotopic compositions of water transpired by leaves relative to soil evaporated vapor (Yakir and Sternberg, 2000). In the past however, precise determinations of isotopic compositions of evapotranspiration (δ*^18^O_ET_*), evaporation (δ*^18^O_E_*), and transpiration (δ*^18^O_T_*) have been challenging since measurements of water vapor were difficult to obtain using conventional cold-trapping methods (e.g., Helliker and Ehleringer, 2002; Williams et al., 2004). Recent developments in laser spectroscopy now enable measurements of δ*^18^O* of ambient water vapor (δ*^18^O_a_*), δ*^18^O_ET_* and its components with high temporal resolution in the field (Werner et al., 2012) and bear a novel opportunity to separate evaporative and transpirational fluxes with higher temporal resolution (Dubbert et al., 2013; Wang et al., 2013).

The main goal of this study was to analyze the contribution of the herbaceous layer to ecosystem water cycle and productivity, which was assessed by combining eddy co-variance and chamber based flux-measurement techniques with a novel laser spectrometer. We hypothesize that the herbaceous understory layer, although vulnerable to drought, plays an important role in the water and carbon cycle, and soil water redistribution. We focused on disentangling the inter-seasonal impact of understory vegetation effects on: (i) the ecosystem water and carbon fluxes, (ii) soil evaporation and (iii) the influence of vegetation on rain infiltration. To explicitly account for the heterogeneity created by the patchy tree cover (Moreno et al., 2007) two experimental sites were installed (under the tree crown and in an adjacent open area) containing understory vegetation and bare soil plots.

## Materials and methods

Isotopic compositions are reported here as ratios *R* between the concentrations of rare and common isotopes (*^18^O*/*^16^O*) or expressed as δ-notation, i.e., relative to Vienna Standard Mean Ocean Water (V-SMOW; Gonfiantini, 1978): δ*^18^O* [‰] = ((*R_sample_* − *R*_*V*−*SMOW*_)/*R*_*V*−*SMOW*_) × 1000.

### Study site and experimental design

Measurements were conducted in an open cork-oak woodland (*Quercus sube*r L.) in central Portugal, approximately 100 km north-east of Lisbon (N39°8′17.84″ W8°20′ 3.76″; Herdade de MacHoqueira do Grou). The trees are widely spaced (209 individuals ha^−1^) with a *LAI* of 1.05 and a gap probability of 0.7 (Piayda et al., unpublished results). The oak trees are managed for cork production and were planted approximately 50 years ago.

The herbaceous layer is dominated by native annual forbs and grasses (see Table 1 for detailed species composition). The site is characterized by Mediterranean climate, with 30 year long-term mean annual temperature of approximately 15.9°C and annual precipitation of 680 mm (Instituto de Meteorologia, Lisbon). We established two sites: one directly under the oak crown projected area and another one in an adjacent open area, 5–7 m distant from any canopy cover. Two types of plots (sized 40 × 80 cm) were installed in each site: bare soil plots with total exclusion of above-ground biomass and root in-growth by inserting trenching meshes (trenching depth = 60 cm; mesh diameter < 1 μm, Plastok, Birkenhead, UK), and understory plots with undisturbed herbaceous vegetation (four plots per site and treatment). Both plot types were replicated 4 times at each site and equipped with soil sensors (16 plots, see below), however gas-exchange understory chamber measurements (see below) were only replicated 3 times, due to time limitations (12 plots total). All plots were established 1 year before measurements to minimize effects of disturbance.

**Table 1:** **List of herbaceous species growing in the open and tree plots in 2011**.

**N-fixing forbs**	**Forbs**	**Grasses**
*Ornithopus compressus* L.	*Crepis vesicaria* L.	*Briza maxiama* L.
*Ornithopus sativus* Brot.	*Geranium spec*	*Lolium multiflorum* Lam.
*Trifolium glanduliferum* Boiss.	*Plantago coronopus* L.	*Vulpia bromoides* Gray
*Trifolium michelianum* Savi	*Rumex acetosella* L.	*Vulpia geniculata* Link
*Trifolium incarnatum* L.	*Silene gallica* L.	
*Trifolium subterraneum* L.	*Spergula arvensis* L.	
*Trifolium resupinatum* L.	*Tolpis barbata* (L.) Gaertn.	
*Trifolium vesiculosum* Savi	*Tuberaria guttata* (L.) Fourr.	

To assess the impact of the understory to ecosystem carbon and water cycling a combination of continuous (i.e., eddy co-variance, environmental sensors, soil profiles) and non-continuous (i.e., chamber and laser based gas-exchange and isotopic and understory biomass observations) measurements were conducted. At the understory level *ET* partitioning could be done on 26 days at the open and 22 days at the tree site and *NEE* partitioning on 23 days at the open and 20 days at the tree site. Measurements were distributed over four measurement campaigns in spring (7.April—3.May), late spring (23.May—16.June), summer (11.—23.September), and fall (23.October—22.November). During winter no measurements were obtained due to strong temperature limitation and consequently very low net water and carbon fluxes. At the ecosystem level partitioning could be achieved for days when our understory field site was within the footprint of the eddy co-variance system and eddy co-variance data was of sufficient quality (i.e., no gap-filled data), which resulted in 9 days equally distributed between spring, summer drought, and fall. Separation of *ET* and *NEE* fluxes was done on diurnal courses repeatedly between 7 a.m. and 7 p.m. (Figure S1) at 5–6 time points, which were used to calculate day-time sums of *ET, E, T* of the understory and the oaks and *NEE, R_eco_*, and *GPP* of understory and oaks. Infiltration of precipitation into the soil on bare soil and vegetated soil patches was estimated for two periods: spring (7. April—16. June) and fall (23.October—22. November).

### Environmental variables and herbaceous biomass

Photosynthetic photon flux density was measured at both sites at approximately 1.5 m height (PPFD, LI-190SB, LI-COR, Lincoln, USA). Rainfall (ARG100 Rain gage, Campbell Scientific, Logan, UT, USA), air temperature, and relative humidity (rH, CS-215 Temperature and Relative Humidity Probe, Campbell Scientific, Logan, UT, USA) were measured and 30 min averages were stored in the datalogger (CR10x, Campbell Scientific, Logan, UT, USA). Soil temperature (custom built pt-100 elements) in 5, 15, 30, and 60 cm depth was measured in vegetation and bare soil plots at both sites and 60 min averages were stored in a datalogger (CR1000, Campbell Scientific, Logan, UT, USA; 4 sensors per depth and treatment). Temperature at the soil surface was manually measured on each measurement day in diurnal cycles corresponding with the gas exchange measurements using temperature probes (GMH 2000, Greisinger electronic, Regenstauf, Germany). Volumetric soil water content (θ_*s*_, 10hs, Decagon, Washington, USA) in 5, 15, 30, and 60 cm depth was measured in vegetation and bare soil plots at both sites and 60 min averages were stored in the datalogger (CR1000, Campbell Scientific, Logan, UT, USA; 4 sensors per depth and treatment). The total water infiltration following each rain event (>2 mm d^−1^) into the upper 60 cm of the soil profile was calculated from θ measurements. Therefore, the maximum increase in θ (m^3^ m^−3^) following a rain event was estimated for each depth separately. The 10 hs sensors integrate over 10 cm soil profile, thus the estimated infiltration (= increase in θ) was representative for the sensors in 5, 15, 30, and 60 cm for 0–10, 10–20, 25–35, and 55–65 cm, respectively. The increase of θ/infiltration in the intermittent depths that were not measured was linearly integrated. Finally, total infiltration into the upper 60 cm of the soil profile was estimated as a sum of all depths and converted to mm d^−1^.

Aboveground biomass of living herbaceous plants was determined destructively on five 40 × 40 cm plots per site randomly selected near the permanent plots. Harvesting took place at six measuring dates: four in spring and two in November. All aboveground parts of living plants were collected, dried (60°C, 48 h) and weighed.

### Eddy-covariance measurements

An ecosystem eddy-covariance flux tower was set up, equipped with a Gill R3A-50 ultrasonic anemometer (Gill Instruments Ltd., Lymington, UK). The tower was equipped with a LI-7000 closed path CO_2_/H_2_O analyzer (LI-COR, Lincoln, USA). The measurement height was about 23.5 m above ground and the tower was in 100 m distance of the experimental field site.

Data were continuously acquired on a field laptop with the eddy covariance data acquisition and processing software package EddyMeas (Meteotools, Jena, DE, Kolle and Rebman, 2007) and are post-processed using EddySoft according to an extended FLUXNET procedure. Heat and water fluxes are corrected for the energy balance closure gap according to Mauder et al. (2013). The fiux gap-filling was made according to Reichstein et al. (2005). Gaps were only filled up to a maximum gap length of 6 days (Piayda et al., 2014).

### Cavity ring-down spectrometer based measurements of δ*^18^O_E_* and understory δ*^18^O_ET_*, and gas-exchange flux measurements

Water and carbon dioxide fluxes and isotopic composition of water fluxes were measured using a Cavity Ring-Down Spectrometer (CRDS, Picarro, Santa Clara, USA) and a CO_2_ infrared gas analyzer (BINOS100; Fisher-Rosemount GmbH & Co., Hasselroth, Germany) in combination with custom built soil chambers. We used 2 chambers that were switched between plots for measurements, following the design of Pape et al. (2009), in an open gas exchange system (n = 3 plots per treatment and experimental site; 12 plots in total). The transparent Plexiglas soil chamber had a total volume of 60 L. The flow through the chamber was regulated as described in Pape et al. (2009) using a fan inside the inlet sampling tube and could be adjusted between 0 and 40 L min^−1^.

All measurements were conducted in diurnal courses with a duration time of roughly 1.5–2 h per measurement point and 5–6 measurement points between 7 a.m. and 7 p.m. (Figure S1). To conduct each measurement point the two chambers were rotated randomly on 6 plots of one experimental site. To calculate net CO_2_ exchange (*NEE*) and evapotranspiration (*ET*) as well as its isotopic composition, background air going into the chamber (at 1.5 m height) and sampling air (coming out of the chamber) were alternately measured. After stable values were reached the final 5 min interval average was used for the calculation of *NEE* and *ET*. Including the time needed to reach stable values, the total duration of the chamber for one measurement point on each plot was between 10 and 15 min. Fluxes of *NEE, ET* as well as total conductance (*g_t_*) were calculated with the gas-exchange equations of Von Caemmerer and Farquhar (1981).

Oxygen isotope compositions of soil evaporation (bare soil plots) as well as evapotranspiration of the understory (vegetation plots) were estimated using a mass balance approach (Dubbert et al., 2013, 2014a):

(1) $$\begin{array}{l} \delta_{E}=\frac{u_{out}w_{out}\delta_{out}-u_{in}w_{in}\delta_{in}}{u_{out}w_{out}-u_{in}w_{in}}\\ \;=\frac{w_{out}\delta_{out}-w_{in}\delta_{in}}{w_{out}-w_{in}}-\frac{w_{in}w_{out}\left(\delta_{out}-\delta_{in}\right)}{w_{out}-w_{in}} \end{array}$$

where *u* is flow rate [mol(air) s^−1^], *w* is mole fraction [mol(H_2_O) mol(air)^−1^] and δ is isotope ratio of the incoming (*in*) and outgoing (*out*) air stream of the chamber. Flow rates are measured with humid air so that conservation of dry air gives *u_in_*(1−*w_in_*) = *u_out_*(1−*w_out_*), which leads to the second line of Equation (1). The second term in Equation (1) corrects for the increased air flow in the chamber due to addition of water by transpiration. In addition to isotopic signatures of soil evaporation and understory evapotranspiration, the oxygen isotope signatures of ambient water vapor (in 9 m height) were measured with the CRDS.

### Sampling and measurement of δ*^18^O* of soil water and precipitation

Soil samples for water extraction and δ*^18^O* analysis were taken on vegetation and bare soil plots using a soil corer on 17 and 15 days at the open and tree site, respectively (see Table S1 for details). Samples were collected from the soil surface (0–0.5 cm depth), 2, 5, 10, 15, 20, and 40 cm soil depths (n = 4 per depth and treatment). Soil water samples were extracted on a custom build vacuum line by cryogenic distillation. Precipitation samples were collected roughly each week. Water δ*^18^O* analysis was performed by headspace equilibration on an Isoprime IRMS (Elementar, Hanau, Germany) coupled via open split connection to a μ gas autosampler (Elementar, Hanau, Germany). Equilibration with 5% He and 95% CO_2_ gas was done for 24 h at 20°C. For every batch of 44 samples 3 different laboratory standards were analyzed. Laboratory standards were regularly calibrated against VSMOW, SLAP, and GISP water standards (IAEA, Vienna). Analytical precision was < 0.1‰.

### Calculation of δ*^18^O* of soil evaporation

Oxygen isotope signatures of soil evaporation were calculated using the Craig and Gordon equation (1965):

(2) $$R_{E}=\frac{1}{\alpha_{k}\alpha^{+}(1-h)}\left(R_{e}-\alpha^{+}hR_{a}\right)$$

where *R_E_* is the isotope ratio (*^18^O*/*^16^O*) of evaporated water vapor and *R_e_* is the isotope ratio of bulk soil water at the evaporating sites. The evaporating site is the vapor-liquid interface below which liquid transport and above which vapor transport is dominant (Braud et al., 2005). It has been shown for unsaturated soils that this site is related to a strong enrichment in soil water isotopic composition relative to the rest of the soil column and an exponential depletion in isotopic signature within few cm of the underlying soil due to evaporative enrichment of the remaining liquid water (Haverd and Cuntz, 2010; Dubbert et al., 2013). Thus, for *R_e_* and temperature at the evaporating sites (*T_e_*), temperature (see Environmental Variables and Herbaceous Biomass) and oxygen isotope signatures of bulk soil water (see Sampling and Measurement of δ*^18^O* of Soil Water and Precipitation) were measured along the soil profile and those values along the soil profile were used where the strongest enrichment in bulk soil δ*^18^O* could be detected (residual soil water volumetric content was only 1% and therefore neglected). Bulk soil δ*^18^O* was estimated with higher resolution along the soil profile than temperature (compare Section Environmental Variables and Herbaceous Biomass and Sampling and Measurement of δ*^18^O* of Soil Water and Precipitation), so in case the highest enrichment in bulk soil δ*^18^O* was found in a depth where temperature was not measured, linear interpolations of the adjacent values were used. In cases, where bulk soil δ*^18^O* was not analyzed for specific dates where gas-exchange data was available and partitioning was conducted, values from adjacent sampling dates were taken. *R_a_* is the isotope ratio of ambient water vapor, α_*k*_ is the kinetic fractionation factor, α^+^ is the water vapor equilibrium fractionation factor (α_*k*_ and α^+^ > 1; Majoube, 1971; Merlivat, 1978; for the formulation of α_*k*_ = α*^nk^_diff_* see Mathieu and Bariac, 1996), and *h* is the relative humidity normalized to *T*_*e*_.

Although direct estimates of *E* and δ*^18^O_E_* were available for bare soil plots, vegetation depresses *E*, and also influences δ*^18^O_E,_* for example due to different isotopic signatures of soil water and also temperature at bare soil and vegetated soil patches (see Table S1 and Dubbert et al., 2013). Therefore, bare soil plots only served to validate the Craig and Gordon equation, because on bare soil plots *E* contributes entirely to the evaporative flux and could be tested against modeling results. Validation was done site specifically, using measured and modeled δ*^18^O_E_* of 26 and 22 diurnal cycles obtained between 7. April and 22. November 2011 at the open and tree site, respectively. Finally, the Craig and Gordon equation was used to calculate δ*^18^O_E_* of vegetation plots.

### Modeling δ*^18^O* of plant leaf water at the evaporating sites and transpiration

To calculate δ*^18^O_T_*, in a first step the isotopic composition of leaf water at the evaporating sites (δ*^18^O_E_*) was calculated. We used the iterative solution of the ordinary differential equation for leaf water at the evaporating sites in non-steady state as in Dongmann et al. (1974; see also Cuntz et al., 2007):

(3) $$R_{e}(t+dt)=R_{c}+\left(R_{e}(t)-R_{c}\right)e^{-\frac{g_{t}w_{i}}{\alpha_{k}\alpha^{+}V_{m}}dt}$$

where *R_e_* (*t* + *dt*) and (*t*) are the isotope ratios of leaf water at the evaporating sites at time *t* and after a time step at time *t* + *dt, g_t_* is the total conductance (mol m^−2^ s^−1^), *w_i_* is the mol fraction in the stomatal cavity, and *V_m_* the mesophyll water volume (mol m^−2^). *R_c_* is the Craig and Gordon steady-state isotope ratio at the evaporating sites, i.e., Equation (1) rearranged for *R_e_* with *R_E_* = *R_x_*, and *R_x_* being the isotope ratio of xylem/source water. We were not able to sample xylem water in large sample sizes, due to methodological restrictions related to the size and lacking lignifications of the herbaceous plant species. Therefore, the source/xylem isotopic ratio was estimated by assuming root water uptake proportional to root density, which was estimated as root biomass (g) per kg soil along the soil profile. In very dry soil conditions this method could pose some error since plants can shift water uptake into deeper, wetter soil layers. However, non-woody species, such as the understory vegetation in this study, have shallow root systems, and therefore lack high ability to shift water uptake depths (Otieno et al., 2011). For further details see Dubbert et al. (2013). Knowing the isotopic signature of leaf water at the evaporating sites, the isotopic signature of plant transpiration can finally be calculated using the Craig and Gordon formulation (Equation 2) with the isotopic signature of leaf water at the evaporating sites in the non-steady-state as *R_e_*.

### Water and carbon partitioning

The contribution of *T* to *ET* at the herbaceous understory scale, *ft* = *T*/*ET*, can be estimated based on measured understory δ*^18^O_ET_* and modeled soil δ*^18^O_E_* and herbaceous δ*^18^O_T_* (Moreira et al., 1997; Yakir and Sternberg, 2000):

(4) $$ft=\frac{\delta^{18}O_{ET}-\delta^{18}O_{E}}{\delta^{18}O_{T}-\delta^{18}O_{E}}$$

This approach is based on the assumption that the isotopic signature of evapotranspiration is a mixing ratio of not more than the two sources (evaporation and transpiration) and that no water vapor is lost other than by the mixing of the two sources with the atmospheric pool (i.e., no condensation).

At the understory level, strong heterogeneity between understory vegetation growing under the tree crown and in open areas was found regarding species development and net fluxes of CO_2_ and water. It is important to account for this heterogeneity when we want to separate understory flux components from net ecosystem carbon or water fluxes. Therefore, an average flux of understory transpiration, soil evaporation, and *NEE* was calculated as:

(5) $$F=P_{gap}F_{open}+\left(1-P_{gap}\right)F_{tree}$$

where *F* denotes the water or carbon flux per m^2^ ground area, the subscripts *open* and *tree* denote the open and the tree site, respectively. *P_gap_* is the canopy gap fraction modeled from the daily course of sun inclination angle and the view zenith angle distribution of *P_gap_* (Piayda et al., unpublished).

At the whole ecosystem level, *ET* was separated into transpiration of cork-oak trees (*T_o_*) by subtracting estimates of understory evapotranspiration measured with the CRDS.

The partitioning of the net CO_2_ fluxes (*NEE)* into gross primary production (*GPP*) and ecosystem respiration (*R_eco_*) followed Lasslop et al. (2010). *GPP* of the understory was estimated by subtracting *R_eco_* from chamber based estimates of understory *NEE*, arguing that *R_eco_* is mainly comprised of heterotrophic soil respiration and root respiration during daytime. This assumption was validated by a comparison of *R_eco_* of the ecosystem tower with *R_eco_* of a nearby understory tower, measuring a very comparable understory community. The *R_eco_* estimates of both towers were in the same range and correlate very well (data not shown).

Water use efficiency (*WUE*) at ecosystem and understory level was calculated. Since changes in *WUE* due to water limitations are often obscured by changes in *VPD* the inherent *WUE* (*iWUE*; Beer et al., 2009) was calculated as:

(6) $$iWUE=\frac{-NEE\;\cdot \;VPD}{ET}$$

At plant level inherent *WUE* was calculated as follows:

(7) $$iWUE=\frac{-GPP\;\cdot VPD}{T}.$$

### Statistical analysis

If not indicated otherwise, all results are presented as mean values with SE (n = 3 – 4). In the case of diurnal cycles, all values of one treatment were integrated into a mean value that was conducted within one measurement point of roughly 1.5 h. In the case daytime sums are presented, these were estimated for each plot replicate and then averaged.

Mann-Whitney *U*-tests were used to examine significant site-specific differences at each measurement day regarding PPFD, soil moisture, soil temperature, understory evapotranspiration, and net carbon exchange (and their components), conductance and oxygen isotope compositions within the ecosystem. Spearman Rank order correlations were used relating ecosystem *ET* and *NEE* components and environmental factors. Non-linear regressions were performed to relate rainfall amount with infiltration difference between vegetation and bare soil plots and relating volumetric soil water content with difference in *iWUE* on understory level and *iWUE* of understory plants. Statistical analyses were carried out with Statistica (Statistica 6.0, StatSoft, Inc., Tulsa, USA).

## Results

### Environmental conditions and net ecosystem carbon and water fluxes

**Figure 1:**
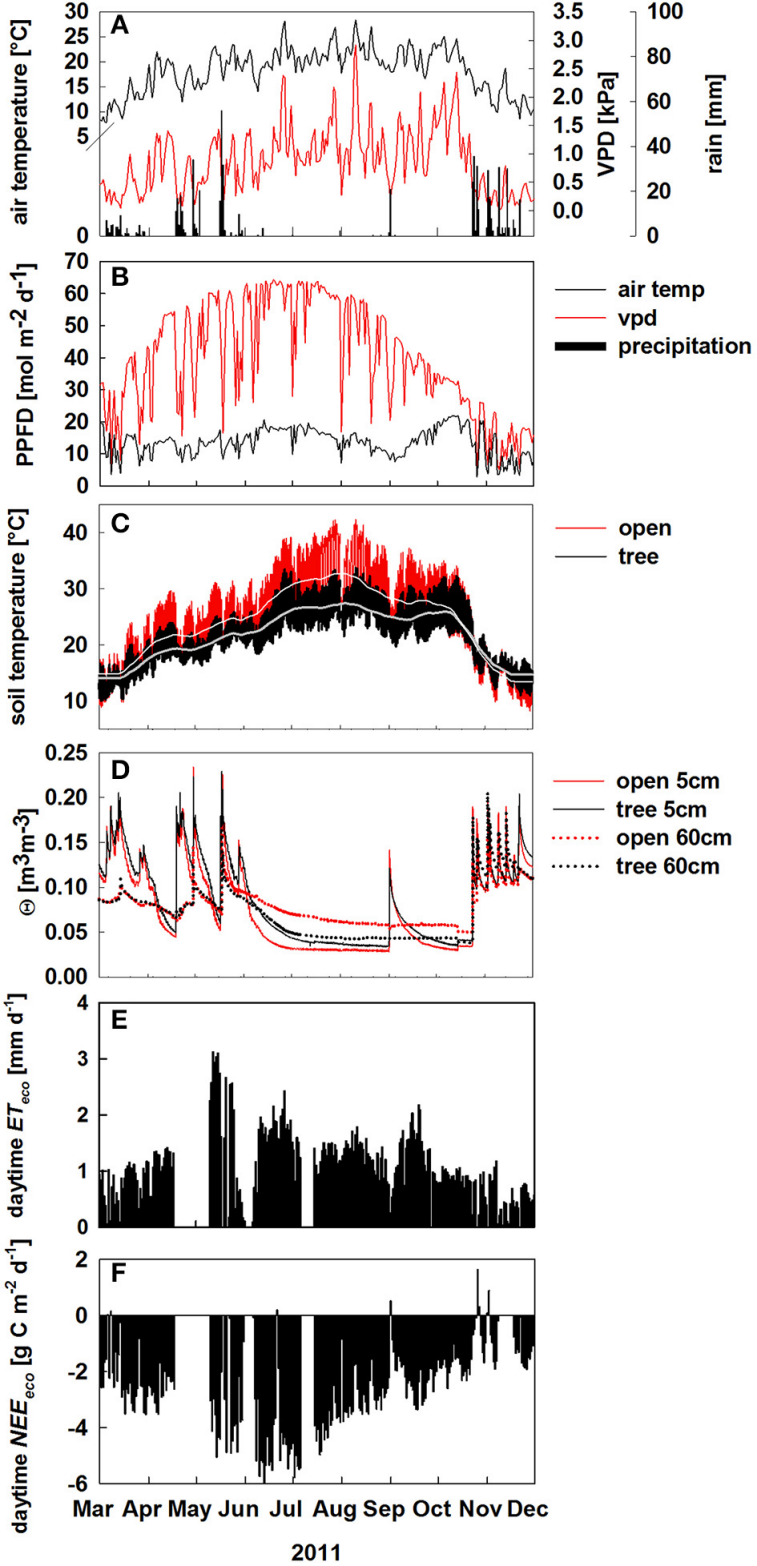
**Environmental conditions from March to December 2011**. **(A)** Daily averages of air temperature (black solid line, °C), vapor pressure deficit (VPD; red solid line, kPa) and daily sums of precipitation (black bars, mm d^−1^); **(B–D)** Environmental conditions at the open (red) and tree site (black) of: daily sums of photosynthetic photon flux density (PPFD; mol m^−2^ d^−1^), hourly values of soil temperature in 5 cm soil depth (lighter lines denote running averages), soil volumetric water content (Θ, m^3^ m^−3^) in 5 and 60 cm soil depth. **(E,F)** daytime integrated net ecosystem fluxes of: evapotranspiration (*ET*, mm d^−1^, black bars), **(E)** and net CO_2_ exchange (*NEE*, g C m^−2^ d^−1^), **(F)** from March to December 2011.

Over the course of the study period, air temperature, and PPFD followed the typical Mediterranean climate pattern (Figure 1). With a total annual rainfall of 800 mm, 2011 was rather wet compared to the long term 30 years mean of 680 mm. Despite high winter precipitation, we observed a first drought period between 1. and 18. April with soil water content (θ) dropping below 0.05 m^3^ m^−3^ (Figure 1D).

Between April and October microclimate conditions differed considerably in the open and under the tree crown: light intensity and soil temperature were reduced by the tree shadow by up to 45 mol m^−2^ d^−1^ (Figure 1B) and up to 7°C (Figure 1C), respectively. Further, θ in 60 cm soil depth was 0.03 m^3^ m^−3^ lower at the tree site during the summer drought compared to the open site (June—October, Figure 1D).

Daytime ecosystem evapotranspiration *ET* reached maximum values in May and declined constantly thereafter (Figure 1E). Likewise, net ecosystem CO_2_ exchange (*NEE)* exhibit strong seasonal changes reaching maximum uptake rates in June (Figure 1F). Notably, the ecosystem was a net carbon sink between March and December 2011 (Figure 1F), although daytime *NEE* declined during summer drought by about 40%. There were only few days, where *NEE* showed a net CO_2_ release during daytime which correspond either to heavy rain events on dry soils resulting in increased soil respiration (“Birch effect,” see Unger et al., 2012) or low photosynthetic uptake on very cloudy days between September and November (Figures 1D,F).

### Vegetation effects on rainfall infiltration

**Figure 2:**
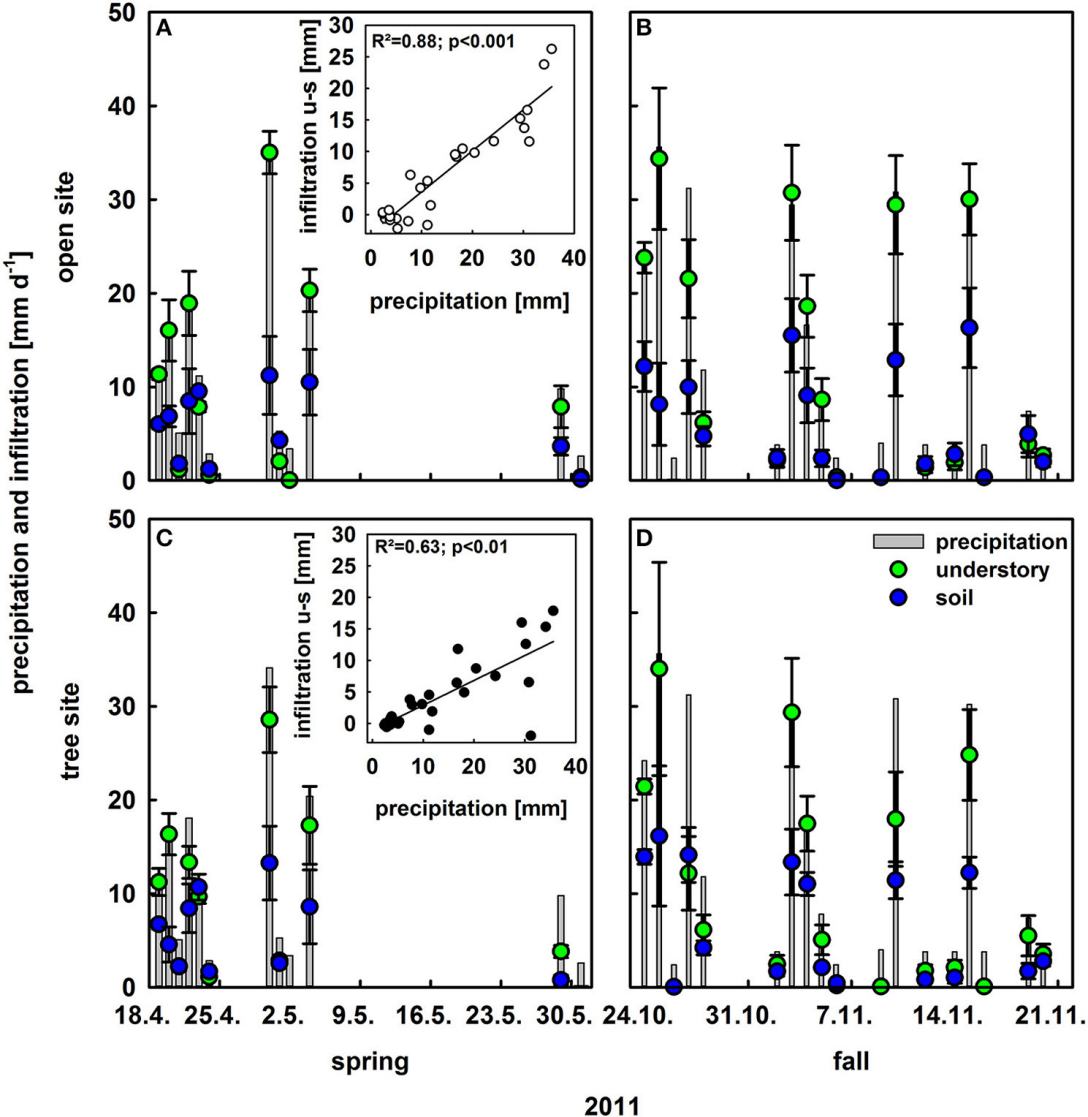
**Infiltration into the soil following rain events >2 mm on understory plots (green bars) and bare soil plots (blue bars; *n* = 4, mean values ± SE) as well as daily precipitation (gray bars) in [mm d^−1^]**. Upper row **(A,B)** is at the open site and lower row **(C,D)** on the tree site; left column **(A,C)** is in spring and right column **(B,D)** in fall. Insets present infiltration differences between understory and bare soil plots vs. precipitation at the open and tree sites with regression lines, coefficients of regression and *p*-values.

To investigate the effect of understory vegetation on rain infiltration, maximum infiltration per rain event was calculated for the open and tree site for bare soil and the understory vegetation plots (Figure 2). The relative infiltration averaged over all rain events (> 2 mm d^−1^) was much higher on understory than on bare soil patches, 0.75 compared to 0.41. This could be observed for both sites and the tree canopy did not have significant further effects on infiltration (Figure 2). Moreover, a significant relationship could be found between the amount of precipitation and the difference in infiltration between bare soil and understory plots (open site: *R*^2^ = 0.88; *p* < 0.001; tree site: *R*^2^ = 0.63; *p* < 0.001): the stronger the rain event, the bigger was the difference in infiltration between bare soil and understory plots (Figure 2 insets).

### Seasonal development of δ*^18^O* within the ecosystem

Besides the influence on rain infiltration, understory vegetation also contributes to ecosystem water loss via transpiration and for a functional understanding of the development of net ecosystem evapotranspiration (*ET*) we separated between plant transpiration and soil evaporation. At the understory level the dense structure of the herbaceous layer prevented a flux based partitioning approach and stable oxygen isotopes (δ*^18^O*) were used to partition *ET*. This requires the knowledge of δ*^18^O* of water sources within the ecosystem as input parameters for modeling δ*^18^O_E_* and δ*^18^O_T_* (Equation 2).

**Figure 3:**
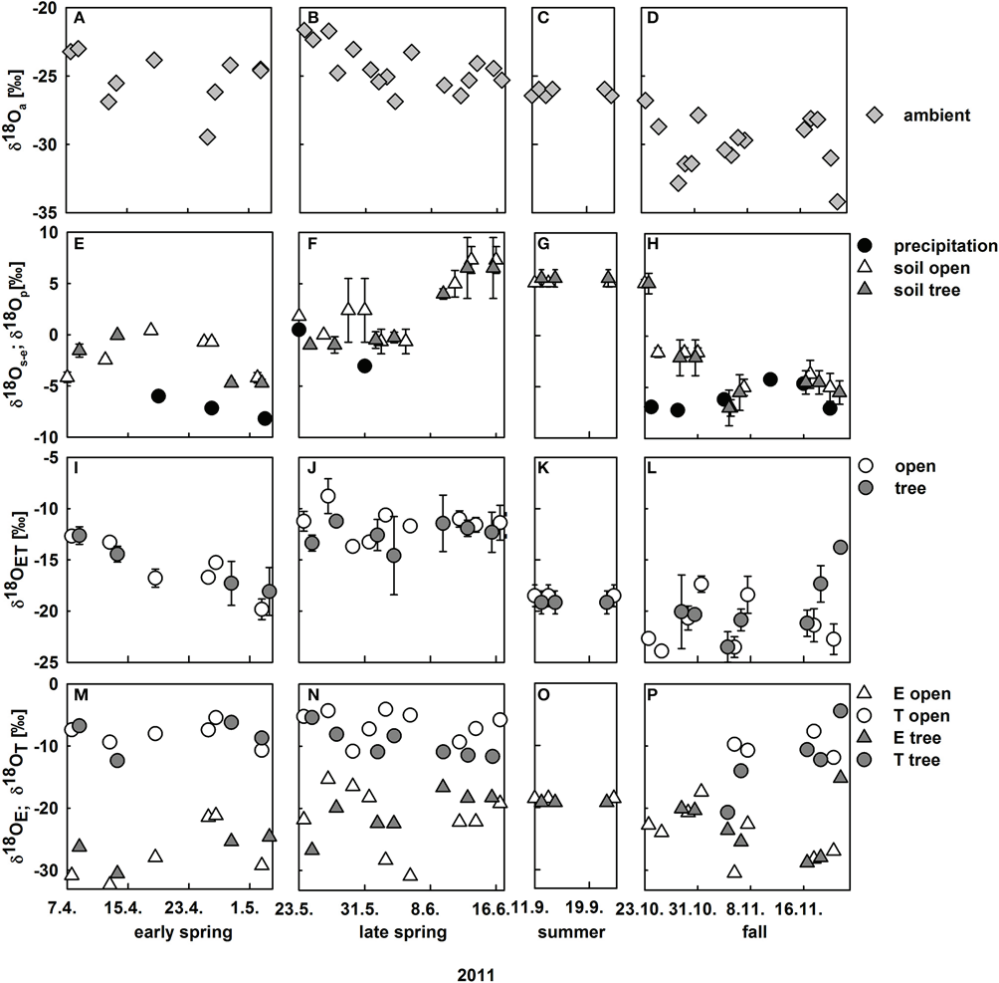
**Development of midday oxygen isotope signatures within the ecosystem from April to November 2011; (A–D)** ambient water vapor δ*^18^O* at 9 m height; **(E–H)** δ*^18^O* of precipitation (black circles) and δ*^18^O* of soil water at the evaporating site on vegetation plots at the open (white triangles) and tree site (gray triangles, mean values ± *SD, n* = 3); **(I–L)** measured δ*^18^O* of evapotranspiration on the open (white circles) and tree site (gray circles, mean values ± *SD, n* = 3); **(M–P)** modeled δ*^18^O* of evaporated vapor from vegetation plots on the open (white triangles) and the tree site (gray triangles) and modeled δ*^18^O* of herbaceous leaf transpired vapor at the open (white circles) and the tree site (gray circles).

Oxygen isotope signatures of ambient vapor (δ*^18^O_a_*) and precipitation (δ*^18^O_p_*)both changed substantially between spring and fall (Figure 3): δ*^18^O_a_* strongly decreased from ca. −25‰ to −30‰ from spring to fall (Figures 3A–D), which can be explained by seasonal changes in the predominant wind direction to north-north-east, delivering more continental, i.e., ^18^*O* depleted, air masses during fall. δ*^18^O_p_* was much higher between −8.2‰ and −0.5‰. In general, oxygen isotope signatures of soil water followed trends in δ*^18^O_p_*. We show the oxygen isotope signatures of soil water from the depth at which highest isotopic enrichment was found, i.e., the isotopic signature of the evaporating front in the soil profile where evaporation occurs (δ^*18*^ *O*_*s*−*e*_) instead of bulk soil δ*^18^O* signatures, because δ^*18*^ *O*_*s*−*e*_ is an important input for the Craig and Gordon equation. δ^*18*^ *O*_*s*−*e*_ was heavily enriched during the summer months compared to precipitation due to much stronger evaporative enrichment during summer and spring as compared to fall (Figure 3; for detailed information on the development of bulk soil δ*^18^O* along the soil profile see Table S1).

Observed midday δ*^18^O* of understory evapotranspiration varied considerably between −8.8‰ and −23.5‰. Notably, variations in δ*^18^O_ET_* were strong between seasons and also within a season (Figure 3I–L). Variations in δ*^18^O_ET_* can be either explained by (i) variation in the relative contribution of component fluxes *E* and *T*, with their differing isotopic signatures or (ii) by change in oxygen isotopic signatures of the component fluxes *E* and *T*. Without the knowledge of the component isotopic signatures this cannot be disentangled. Consequently, these were modeled, based on the isotopic input parameters (see Section Calculation of δ*^18^O* of Soil Evaporation and Modeling δ*^18^O* of Plant Leaf Water at the Evaporating Sites and transpiration).

**Figure 4:**
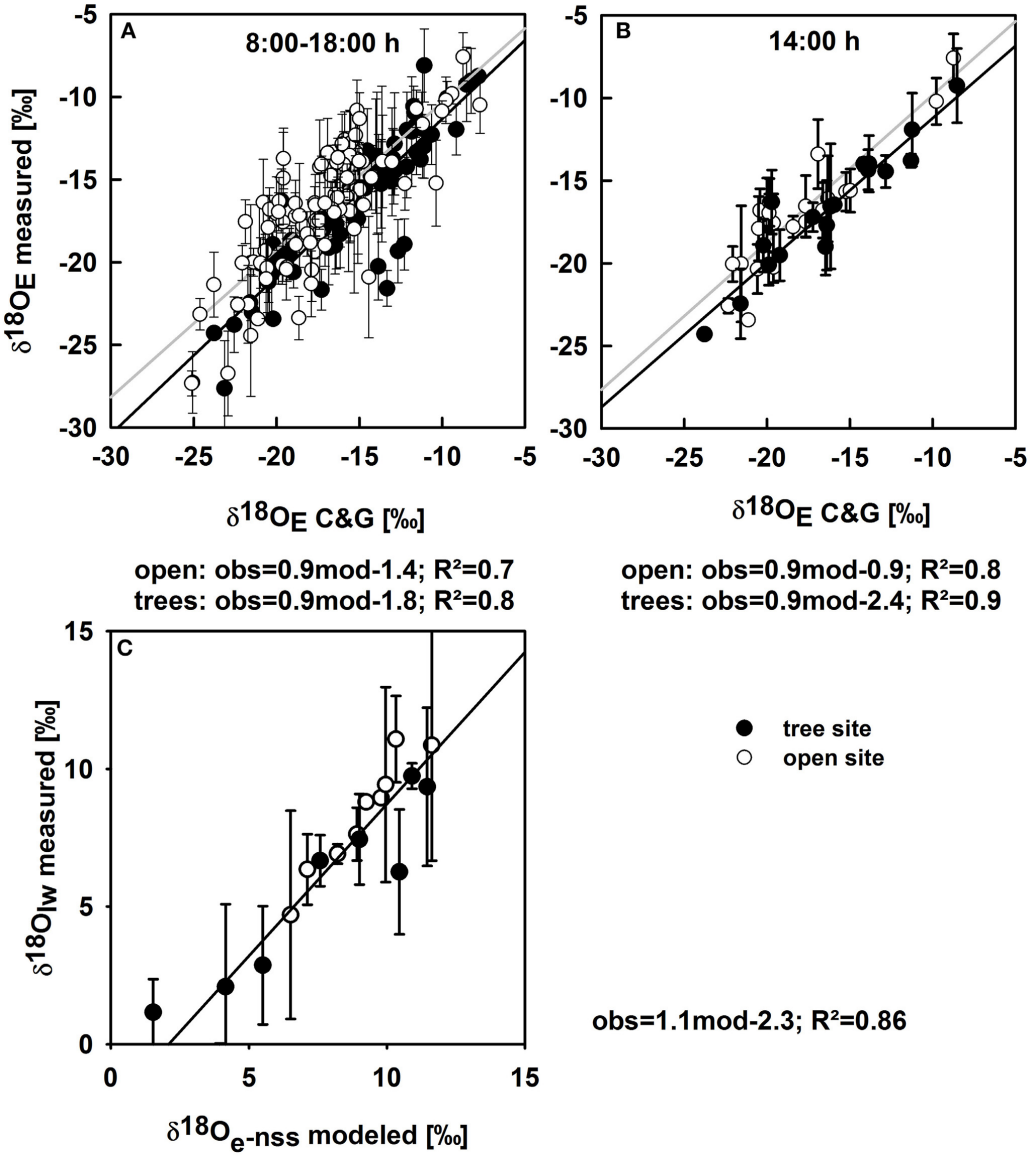
**(A)** Oxygen isotope signatures of soil evaporation on bare soil plots calculated with the Craig and Gordon equation vs. measured values for the open (white circles) and tree sites (black circles) of all measurements (mean values ± *SE*; *n* = 3); the gray and black line denote regression lines for the open and tree sites, respectively. **(B)** Modeled against measured values during midday only (14:00 h). **(C)** Modeled δ*^18^O* of leaf water at the evaporating sites in the non-steady state vs. measured oxygen isotope signatures of bulk leaf water for the open (white circles) and tree site (black circles) for all available data points of measured leaf water δ*^18^O* throughout the study period. Regression equations (observed vs. modeled), correlation coefficients are given below the plots. *p*-values were less than 0.001 for all regressions.

Before the isotope signature of soil evaporation was modeled at vegetated soil patches (Figures 3M–P), the Craig and Gordon equation was tested against direct estimates of δ*^18^O_E_* obtained at bare soil plots, where *E* contributes fully to *ET*: calculated δ*^18^O_E_* is in very good agreement with CRDS based measurements of δ*^18^O_E_* for soil conditions ranging between residual to nearly saturated soil water content (Figure 4A). The agreement between measured and modeled δ*^18^O_E_* was best during midday. However, including morning and afternoon records decreased the coefficient of determination but did not significantly alter the regressions slope and offset (Figure 4).

Modeled midday δ*^18^O_E_* estimated on vegetation plots ranged from −15.1‰ to −31.2‰ and the inter-seasonal development of δ*^18^O_E_* was similar to the development of δ*^18^ O*_*s*−*e*_ (Figures 3M–P).

δ*^18^O_T_* was modeled in two steps, first calculating δ*^18^O* of leaf water at the evaporating sites in the non-steady state (see Section Modeling δ*^18^O* of Plant Leaf Water at the Evaporating Sites and Transpiration and Figure 4C). Modeled δ*^18^O* of leaf water at the evaporating sites was well-correlated with measured bulk leaf water δ*^18^O*, with a negative offset of measured leaf water of 2.3‰ (Figure 4D), owing to the Péclet effect (Farquhar and Lloyd, 1993) as bulk leaf δ*^18^O* contains a mixed signal of non-fractionated xylem water and water at the evaporating sites that is highly enriched in δ*^18^O* (Yakir, 1992). Midday δ*^18^O_T_* ranged between −3.9‰ and −20.1‰ and followed no clear inter-seasonal pattern. Clearly, the strong decrease in δ*^18^O_ET_* from −12.7‰ to −19.8‰ during April was caused by a strong decrease in *T* (Figures 3I,M), while the slight overall increase of δ*^18^O_ET_* in fall can be mainly explained by decreased δ*^18^O_E_* and increased δ*^18^O_T_* (Figures 3L,P).

### Seasonal development of herbaceous ET and NEE components

**Figure 5:**
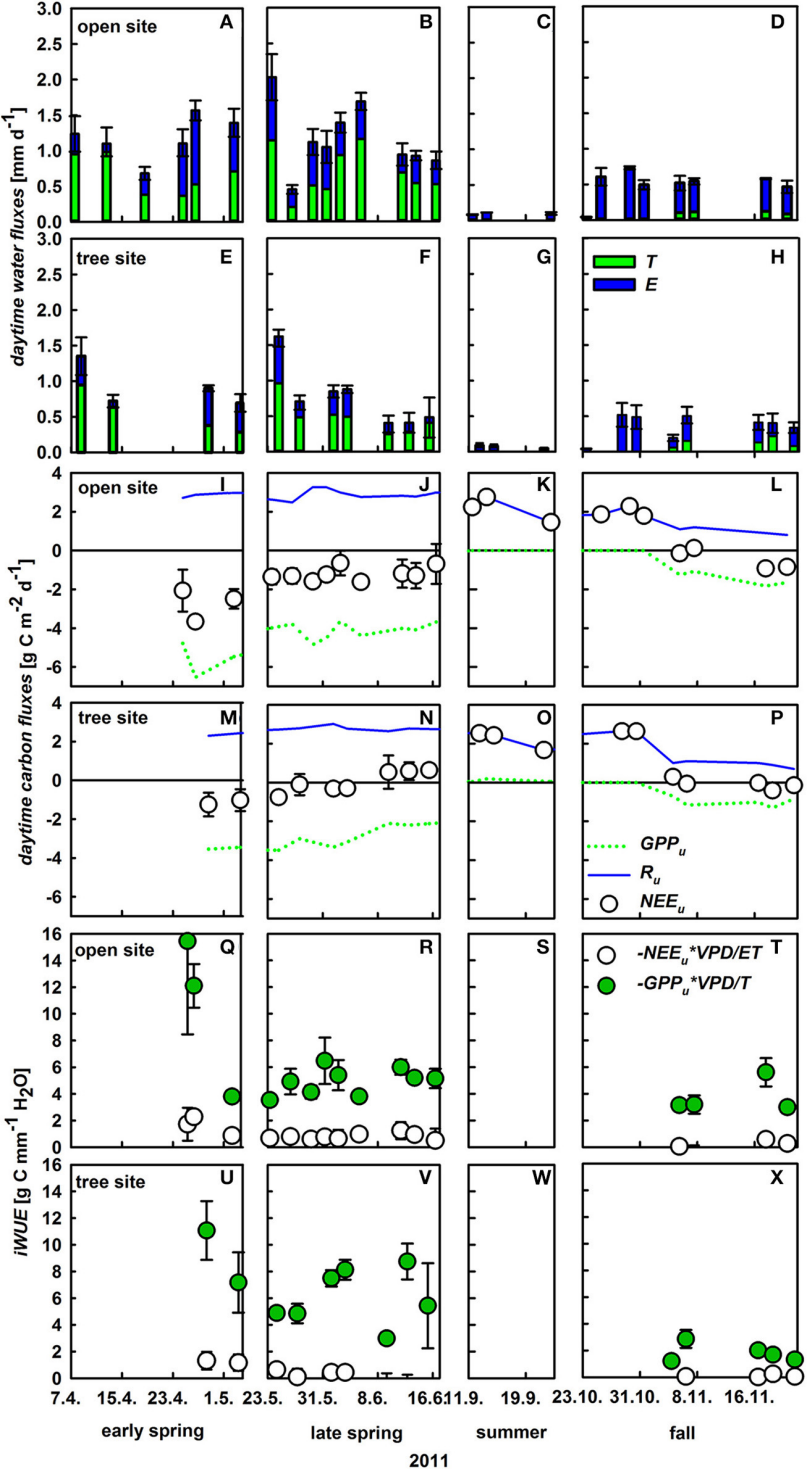
**Daytime integrated understory evapotranspiration (*ET*, mm d^−1^, mean values ± *SD, n* = 3), which is the sum of herbaceous layer transpiration (*T*, green bars) and soil evaporation (*E*, blue bars) at the open (A–D) and tree site (E–H); daytime integrated net understory CO_2_ exchange (*NEE*, g C m^−2^ d^−1^, white circles; mean values ± SE, *n* = 3), herbaceous gross primary production (*GPP*, dashed green line, mean values, *n* = 3) and respiration (*R*, blue line, mean values, *n* = 3) on the open (I–L) and tree site (M–P)**. Inherent water-use efficiency (*iWUE*) of the whole understory (*GPP_u_* × *VPD*/*ET*, white circles), and understory vegetation (*GPP_u_* × *VPD*/*T*, green circles). *iWUE* was calculated from daytime integrated values of *ET, T*, and *GPP_u_* for the open site **(Q–T)** and the tree site **(U–X)**.

Understory *ET* partitioning was based on diurnal observations of understory *ET*, δ*^18^O_ET_* and derived δ*^18^O_T_* and δ*^18^O_E_*, which were used to calculate day-time sums of *ET, E*, and *T* of the understory (Figure S1). Notably, daytime integrated understory transpiration and soil evaporation displayed strong short-term variability (Figures 5A,B,E,F). Within April *T* varied between 0.28 and 0.99 mm d^−1^ at both sites and *E* between 0.07 and 1.04 mm d^−1^ at both sites, respectively and both fluxes were in the same range during spring. Likewise, the relative contribution of *T* to *ET* varied between 34 and 93% between April and June. Understory *ET* was significantly lower at the tree site compared to the open site during the growing season (*U*-test, *p* < 0.05), especially in the transition period between spring and summer (late May to mid-June). This was mainly caused by lower understory transpiration due to a significant lower conductance (Figures 5A–H, Table 2). On an annual basis, herbaceous *T* played a dominant role during the main growing season from April to the onset of summer drought, while soil *E* was equally high during spring and fall (0.4 ± 0.1 mm d^−1^), only ceasing during the summer drought period. Thus, the relative small increase of net understory *ET* in response to increased soil θ in fall was caused by very low *T* (0.12 ± 0.03 mm d^−1^) of the newly established understory vegetation.

**Table 2:** **Daytime mean conductance (mmol m^−2^ s^−1^) of the herbaceous layer during spring, late spring, and fall (mean values ± SE) at the open and tree site**.

**Season**	**Open site**	**Tree site**	***p*-level**
Spring	111.6 ± 35	85.7 ± 41	*n.s*.
Late spring	102.7 ± 47	65.5 ± 48	0.02
Fall	130.2 ± 101	103.8 ± 93	*n.s*.

*Site specific differences are indicated at p < 0.05 (Mann-Whitney U-test)*.

In contrast to ecosystem *NEE, NEE* of the understory (*NEE_u_*) turns to a net carbon source at the onset of summer with net respiration rates of up to 2.8 g C m^−2^ d^−1^ (Figures 5I–P). Understory respiration was relatively stable over the measurement period only declining slightly during fall, due to decreasing temperatures. Hence, variability in *NEE_u_* was mainly triggered by changes in gross primary production of the understory (*GPP_u_*) which peaked in late April at –6.5 g C m^−2^ d^−1^ corresponding to the observed peak in understory aboveground biomass (70 ± 9 and 71 ± 11 g m^−2^ at the open and tree site, Table 3). Die-back of the understory vegetation in late spring and accordingly a decline in *GPP_u_* was responsible for the net release of carbon from the understory during summer, while the germination in late October led to a swift increase of *GPP_u_* up to 1.8 g C m^−2^ d^−1^. Notably, significant site-specific differences were found in *GPP_u_* and *NEE_u_* from late May onward (*U*-test, *p* < 0.05). The die-back of the understory vegetation occurred 2 weeks earlier at the tree site, hence the reduction in *GPP_u_* was stronger under the trees with 50% compared to 20% reduction from late April to mid-June at the open site (Figures 5I–P).

**Table 3:** **Living aboveground biomass (g m^−2^) of the herbaceous layer in spring and fall 2011 on the open and tree site (mean values ± SE; *n* = 5)**.

**Date**	**Open site**	**Tree site**
8.4.2011	69 ± 4	72 ± 21
24.4.2011	70 ± 9	71 ± 11
27.5.2011	67 ± 7	72 ± 23
14.6.2011	33 ± 12	17 ± 9
1.11.2011	14 ± 2	15 ± 4
14.11.2011	43 ± 7	41 ± 7

Inherent water-use efficiency (*iWUE*) was calculated for the whole understory (including respiratory fluxes and soil evaporation) as well as the vegetation level (*GPP* and transpiration only; Figures 5Q–X). Understory *iWUE* did not show a pronounced inter-seasonal development and was 2.2 ± 1.2 at the open and 2.5 ± 1.2 g C mm^−1^ H_2_O at the tree site. Plant level *iWUE* was always higher than understory *iWUE*, however the difference became very pronounced following rain events and a linear relationship could be detected between volumetric soil water content and difference in *iWUE* on understory vs. plant level (*R*^2^ = 0.3; *p* = 0.01).

### Contribution of understory vegetation and soil to the ecosystem carbon and water fluxes

**Figure 6:**
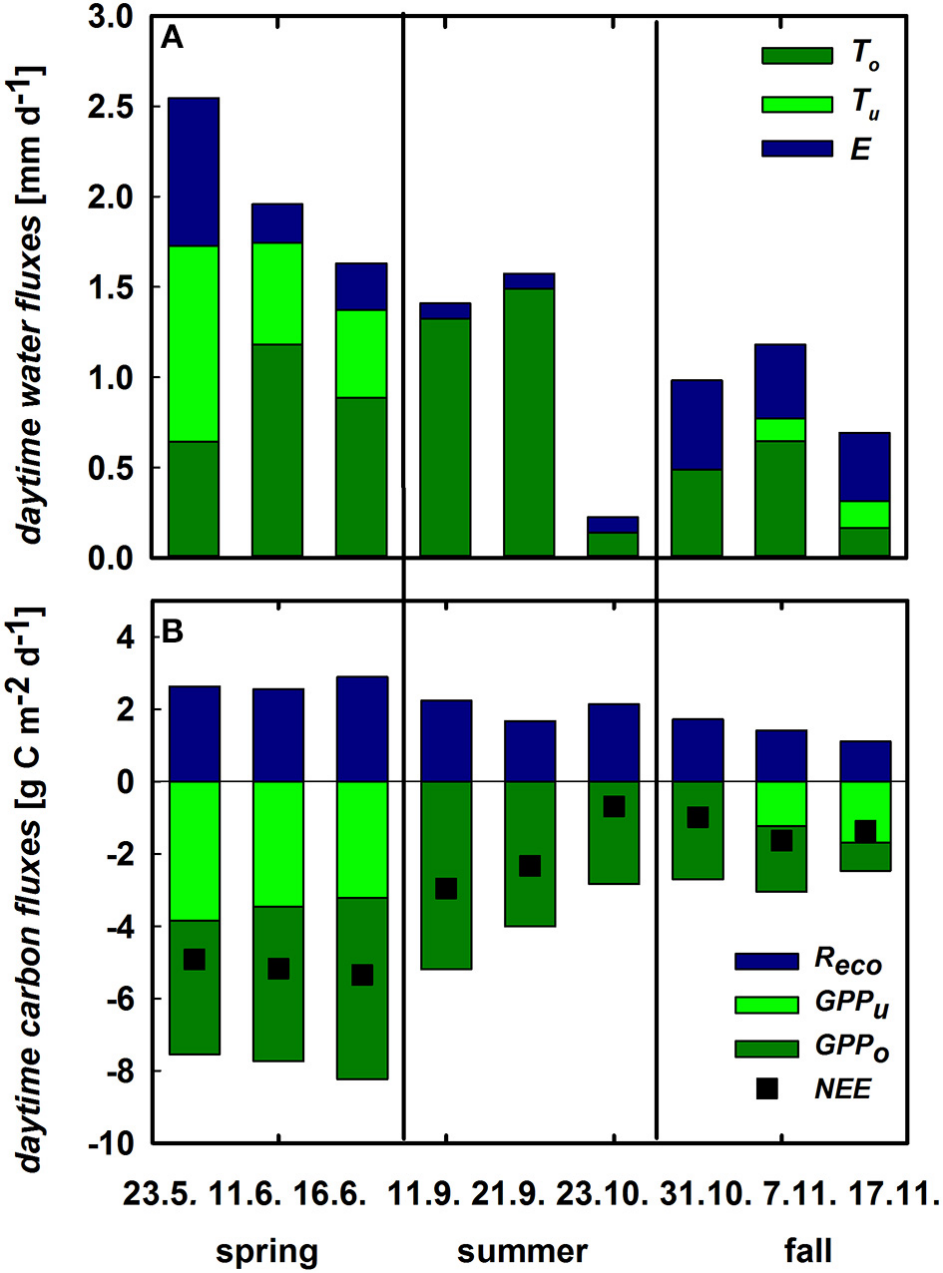
**(A)** Daytime integrated ecosystem evapotranspiration (*ET*, sum of the stacked bars) and its components cork oak transpiration (*T_o_*, dark green), herbaceous transpiration (*T_u_*, green) and soil evaporation (*E*, blue, all mm d^−1^). **(B)** Daytime integrated *GPP* of cork oaks (*GPP_o_*, dark green) and understory (*GPP_u_*, green), ecosystem respiration (*R_eco_*; blue), and net ecosystem CO_2_exchange (*NEE*, black squares, all in g C m^−2^ d^−1^).

The contribution of the understory vegetation to whole ecosystem *ET* was highest during its growth peak in spring. In contrast, soil *E* was the dominant flux of ecosystem *ET* in fall reaching 55% of total *ET*. Herbaceous *T* and soil *E* alike decreased toward the beginning of the summer drought period from 43 and 32% in May to 30 and 16% in June, respectively (Figure 6A), both being negligible for ecosystem *ET* during summer. Likewise, herbaceous *GPP* displayed the highest contribution to ecosystem *GPP* during spring but declining from 51% to 36% toward the onset of summer drought in June. After its germination in fall, understory contribution to *GPP_eco_* increased to 50% within 2 weeks (Figure 6B). Despite the long drought period, cork-oak *GPP* as well as *T* were relatively stable during spring and summer (−4.4 ± 0.65 g C m^−2^ d^−1^ and 1.12 ± 0.14 mm d^−1^, respectively) and were declining only drastically toward the end of summer and remaining low during autumn (–1.8 ± 0.96 g C m^−2^ d^−1^ and 0.2 ± 0.16 mm d^−1^; Figures 6A,B). Since *R_eco_* was relatively stable throughout the year (at 2.1 ± 0.6 g C m^−2^ d^−1^ on average; Figure 6B), changes in ecosystem *NEE*, especially between spring and summer, can mostly be attributed to understory vegetation dynamics.

**Figure 7:**
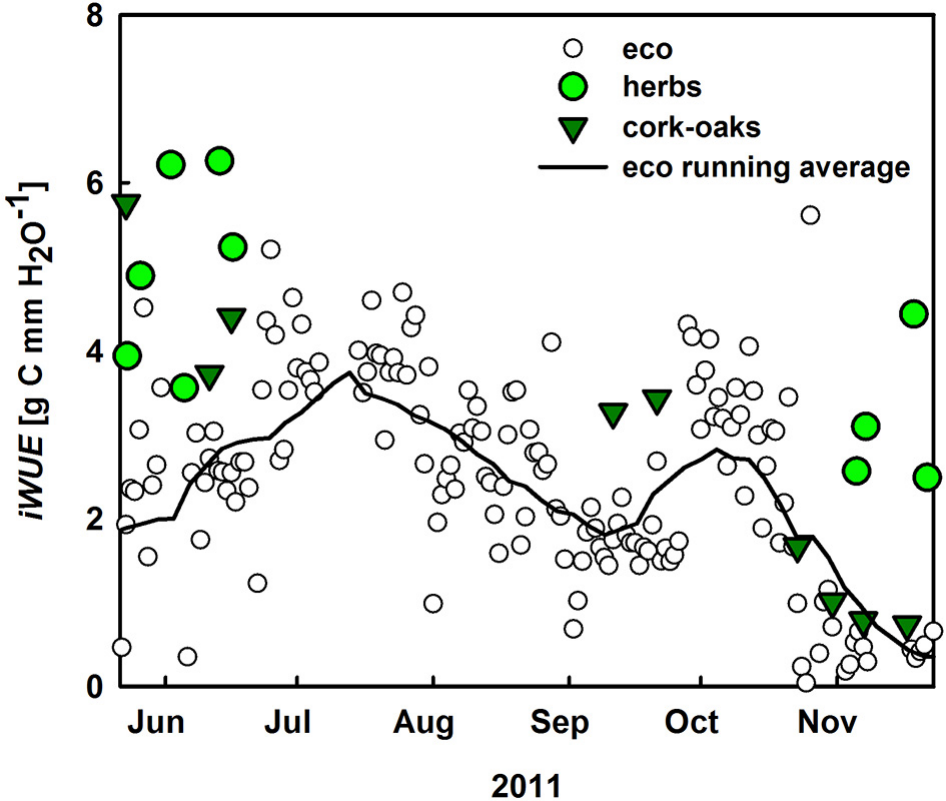
**Inherent water-use efficiency (*iWUE, NEE* × *VPD*/*ET*) of the ecosystem (white circles), the black line represents the running average as well as at plant level (*GPP* × *VPD*/*T*) for cork oaks (dark green) and understory vegetation (green circles)**.

*iWUE* was calculated at ecosystem (–*NEE* × *VPD*/*ET*) and plant level (–*GPP* × *VPD*/*T*; Figure 7). In general, cork-oak *iWUE* was within the range of ecosystem *iWUE*, which increased to 3.8 g C mm^−1^ H_2_O with the onset of summer drought but then steadily declined toward fall. Notably, understory *iWUE* was similar to cork-oak and also ecosystem *iWUE* in spring but much higher in fall (Figure 7).

## Discussion

In semi-arid ecosystems, such as Mediterranean evergreen oak woodlands with sparse tree cover, water is the major factor limiting ecosystem productivity. Future climate change scenarios propose even increased drought and altered precipitation pattern in the Mediterranean (IPCC, 2007; Costa et al., 2012; Jongen et al., 2013). Under these conditions, an efficient use of the limited water supply is crucial (Wang et al., 2012) and advancements of observational methods and modeling approaches are vitally important to better understand vegetation-soil-water feedbacks. We hypothesized that in savanna type ecosystems the herbaceous understory layer, despite its ephemeral life form, plays an important role in the water and carbon balances and for ecosystem resilience toward drought. In the following, this shall be discussed with respect to the contribution of the understory to total ecosystem *ET* and productivity, as well as influence on unproductive soil water loss, i.e., evaporation (*E*) and soil water distribution.

The recent developments in laser spectroscopy enabled us to measure δ*^18^O* of ambient vapor (δ*^18^O_a_*), of understory evapotranspiration (δ*^18^O_ET_*) and its components with a high temporal resolution. The direct observations of δ*^18^O_E_* on bare soil plots allowed a detailed validation of the Craig and Gordon (1965) model for the first time over a whole growing season regarding short time-scales, i.e., differences on a diurnal basis, as well as under extreme conditions (saturated or dry soils; Dubbert et al., 2013; Wang et al., 2013). We could show that calculated δ*^18^O_E_* is in very good agreement with measurements of δ*^18^O_E_* even during early morning and afternoon, where environmental conditions change swiftly. However, a thorough validation of the models estimating δ*^18^O* soil evaporation (Dubbert et al., 2013) and plant transpiration (*T*, Dubbert et al., 2014a) are pivotal. For example, assuming *T* to be in isotopic steady-state leads to offsets of up to 70% in the estimation of the fraction of *T* on total understory evapotranspiration in this ecosystem (Dubbert et al., 2013), exceeding previous uncertainty estimates of around 25% (Yepez et al., 2007). This also indicates that the impact of not considering the effect of non-steady-state transpiration on *ET* partitioning probably differs between plant functional types and ecosystems (see Dubbert et al., 2014a). Similarly, the Craig and Gordon equation is very sensitive to uncertainties in estimates of temperature and oxygen isotope signatures of soil water at the evaporating front (*T_e_* and *R_e_*; see Braud et al., 2005; Rothfuss et al., 2012; Dubbert et al., 2013); hence taking averages of parts of the soil profile, as done by previous studies (Yepez et al., 2005; Lai et al., 2006; Wang et al., 2010), likely leads to large uncertainties not only in the estimate of δ*^18^O_E_* but also in the partitioning (*T*/*ET*).

The coupling of the laser spectrometer to gas-exchange chambers for this isotope based *ET* partitioning approach further offered the opportunity to separate between herbaceous transpiration and soil evaporation for the first time over a whole growing season with a temporal resolution exceeding by far that of previous studies, who mostly were able to estimate *T/ET* for 1 up to 6 days over the growing season (see for comparison Williams et al., 2004; Yepez et al., 2007; Wang et al., 2013; Hu et al., 2014). This has strong potential to enhance our functional understanding of soil evaporation dynamics and vegetation-soil feedbacks within the water cycle, specifically for grassland ecosystems where *ET* can hardly be separated by classical flux based approaches (but see the modeling approach of Hu et al., 2009).

One main observation of this study was the distinct responses of understory *T* and soil *E* to changes in environmental conditions. The small contribution of *T* shortly after a rain pulse is due to the swift increase in soil *E* (Scott et al., 2006; Raz-Yaseef et al., 2012). In contrast, plant *T* strongly decreased upon rain events and only very gradually increased thereafter. During drought, *E* also declined much faster, while plants maintained a relatively stable transpiration rate even under rather dry soil conditions. Raz-Yaseef et al. (2012) explained such findings with the regulation of *T* and *E* by different soil layer θ_*s*_. However, a correlation between *T* and θ_*s*_ of all obtained depths (5, 15, 30, and 60 cm) could not be detected even when θ_*s*_ was low. While *E* was significantly correlated with top soil θ_*s*_ (*R*^2^ = 0.55, *p* < 0.001), a correlation with VPD could only be observed when θ_*s*_ was strongly limiting *E*. By contrast, understory *T* was correlated with *VPD* instead (*R*^2^ = 0.57, *p* < 0.001), highlighting that considering *E* and understory *T* separately is crucial for understanding changes in net *ET*. Moreover, soil evaporation at both sites was correlated with understory biomass development: the higher aboveground biomass the smaller the soil fluxes (see also Barr et al., 2004). Vegetation cover, depending mostly on *LAI*, can largely reduce unproductive soil evaporation (Hu et al., 2009; Wang et al., 2010; Raz-Yaseef et al., 2012). We found up to 40% reduction of *E* on understory vegetation plots compared to bare soil plots (bare soil *E* rates are not shown). Reducing bare soil evaporation has therefore been addressed as a critical issue in many dry-lands (Wang et al., 2012). Averaged for the periods where understory vegetation was present, soil *E* contributed a similar amount to ecosystem *ET* than understory *T* (27 and 29%, respectively), which was largely neglected in previous studies (Paço et al., 2009; Jasechko et al., 2013). However, soil *E* contributed significantly only when water was not limiting plant photosynthesis and growth. By contrast, during times of low water availability, inherent *WUE* increased, which was at least in parts due to strongly decreased soil evaporation rates (Figures 5, 6, Pereira et al., 2007).

Furthermore, comparisons of inherent *WUE* reflecting water limitation effects (Vickers et al., 2012; Eamus et al., 2013) at ecosystem vs. plant scale were conducted. In semi-arid regions *iWUE* often increases in times of moderate drought stress of the vegetation, reflecting the ability of plants to adjust their photosynthetic capacity and/or stomatal control in times of lower water availability (Scott et al., 2006; Yepez et al., 2007; Jongen et al., 2011; Vickers et al., 2012; Eamus et al., 2013), which can be seen at the ecosystem level during early summer (June-July). The decrease in ecosystem and tree *iWUE* over the later course of summer might be explained, on the other hand, by temperature and light stress (Pereira et al., 2006). Trees did not down-regulate their transpiration very strongly until late August (D'Odorico; Figure 6), probably due to their supposed access to deep soil water layers and/or groundwater, but the photosynthetic apparatus might still have been limited by the higher average (leaf) temperatures in summer compared to spring (Werner and Correia, 1996; Werner et al., 2006), thus leading to a decrease in *iWUE* (Pereira et al., 2007). Comparing ecosystem with plant level *iWUE*, a large impact of either soil evaporation or ecosystem respiration on ecosystem *WUE* should be reflected in lower ecosystem scale *iWUE* (–*NEE × VPD/ET*) compared to *iWUE* of cork-oaks and the understory (–*GPP × VPD/T*). While *iWUE* of the cork-oaks was mostly within range of ecosystem *iWUE, iWUE* of the understory plants was higher than ecosystem *iWUE* in both spring and fall. In early spring and fall these smaller values on ecosystem scale were caused by both high soil evaporation and *R_eco_* rates. During late spring however, soil evaporation was small and the lower ecosystem *iWUE* compared to plant *iWUE* was mainly caused by high *R_eco_* rates. This confirms that during times of water limitation ecosystem *iWUE* is not negatively affected by soil *E*. Notably, even the strong differences between ecosystem and plant *iWUE* at the understory scale, immediately following rain events, were caused by a strong increase in plant *iWUE* due to decreased *T_u_* immediately after rainfall and not by a decrease in *iWUE_eco_*. Moreover, the impact of the understory vegetation on ecosystem productivity was as large as its contribution to the water cycle (see also Unger et al., 2009, 2010), leading to similar or even higher *iWUE* of the understory and cork-oaks and, hence, a significant contribution of the understory layer to the ecosystem sink strength in spring and fall.

In addition to this contribution to ecosystem productivity and the reduction of soil evaporation, a third beneficial effect of understory vegetation on ecosystem functioning was identified: understory vegetation impact on soil water infiltration (Tromble, 1988; Dawson, 1993; Schwinning and Ehleringer, 2001; Devitt and Smith, 2002; Bhark and Small, 2003; Huxman et al., 2005; Kurz-Besson et al., 2006; Scott et al., 2014). A positive feedback of vegetation biomass on rain water infiltration is often found in arid ecosystems with open canopies, where it alters spatial distribution and enhances rain use efficiency of the vegetation (Bromley et al., 1997; Couteron and Kokou, 1997; Rietkerk et al., 2002; D'Odorico and Porporato, 2006; Chen et al., 2013). On the other hand, vegetation canopies intercept rainfall, and a substantial proportion of this rainfall interception can be lost due to evaporation from plant surfaces (Tromble, 1988). Here, we observed contrasting effects on rainfall infiltration of the two different vegetation types: the cork-oak canopy had no significant influence on infiltration, while the understory vegetation cover significantly increased infiltration compared to bare soil plots. Bhark and Small (2003) report that this beneficial influence is enhanced in ecosystems with strong natural surface run-off on bare soils with reduced hydraulic conductivity due to a sealed soil layer during the dry period (Chen et al., 2013), which is the case at our study site. Notably, a significant relationship between rain fall intensity and differences in infiltration between bare soil and understory patches could be observed (Bhark and Small, 2003). Likewise, Thompson et al. (2010) found an increasing effect of vegetation biomass on infiltration with decreasing soil water availability. Therefore, the presence of a fully developed herbaceous layer should be even more important with increased drought.

Moreover, herbaceous understory vegetation has been shown to facilitate tree growth and fruit production by increasing soil N (Pulido et al., 2010; Rolo and Moreno, 2011). It can be expected that repeated plowing, liming and sowing of a legume rich seed mixture, a common practice in agro-silvo-pastoral systems in Portugal also done in a 3–5 year interval at our site, significantly increases the contribution of N-fixing species intensifying this effect (Crespo, 2006).

Finally, the understory vegetation itself is highly vulnerable to drought, which is underlined by the significantly earlier die back of the understory vegetation under the trees compared to open areas at the onset of summer drought, when environmental stress increased. This earlier senescence below the tree canopy predominantly affected N-fixers and grasses and suggests competition with oak trees for water from the top soil layers as also herbaceous vegetation transpiration and conductance were significantly reduced by 40% and 45%, respectively (see also Moreno, 2008; Dubbert et al., 2014b). This drought induced competition even influenced total ecosystem sink strength in spring, as it reduced the overall understory productivity on average by 22% on the tree compared to the open sites during the last 3 weeks of the herbaceous vegetation period.

In conclusion, beneficial understory vegetation effects were dominant, as herbaceous biomass strongly increased rain infiltration, diminished soil *E* and significantly added to the ecosystem carbon sink strength. However, the observed vulnerability of the understory vegetation to drought and competition for water with trees suggests, that increased drought and altered precipitation pattern as predicted in future climate change scenarios for the Mediterranean basin not only threaten understory development. They also very likely decrease rain infiltration and ground water recharge by decreasing understory vegetation cover and increasing amount of heavy precipitation events with high run-off from sealed bare soils. This in turn can severely diminish cork-oak productivity and hence the resilience of the ecosystem toward drought (Scott et al., 2014).

### Conflict of interest statement

The authors declare that the research was conducted in the absence of any commercial or financial relationships that could be construed as a potential conflict of interest.
