# Supplementary material for: Stable oxygen isotope and flux partitioning demonstrates understory of an oak savanna contributes up to half of ecosystem carbon and water exchange
Source: Front Plant Sci. 2014 Oct 7;5:530. doi: 10.3389/fpls.2014.00530 (PMC4188126; doi:10.3389/fpls.2014.00530)
Supplement: Supplementary file 1 [file DataSheet1.DOCX]

**Supporting information**


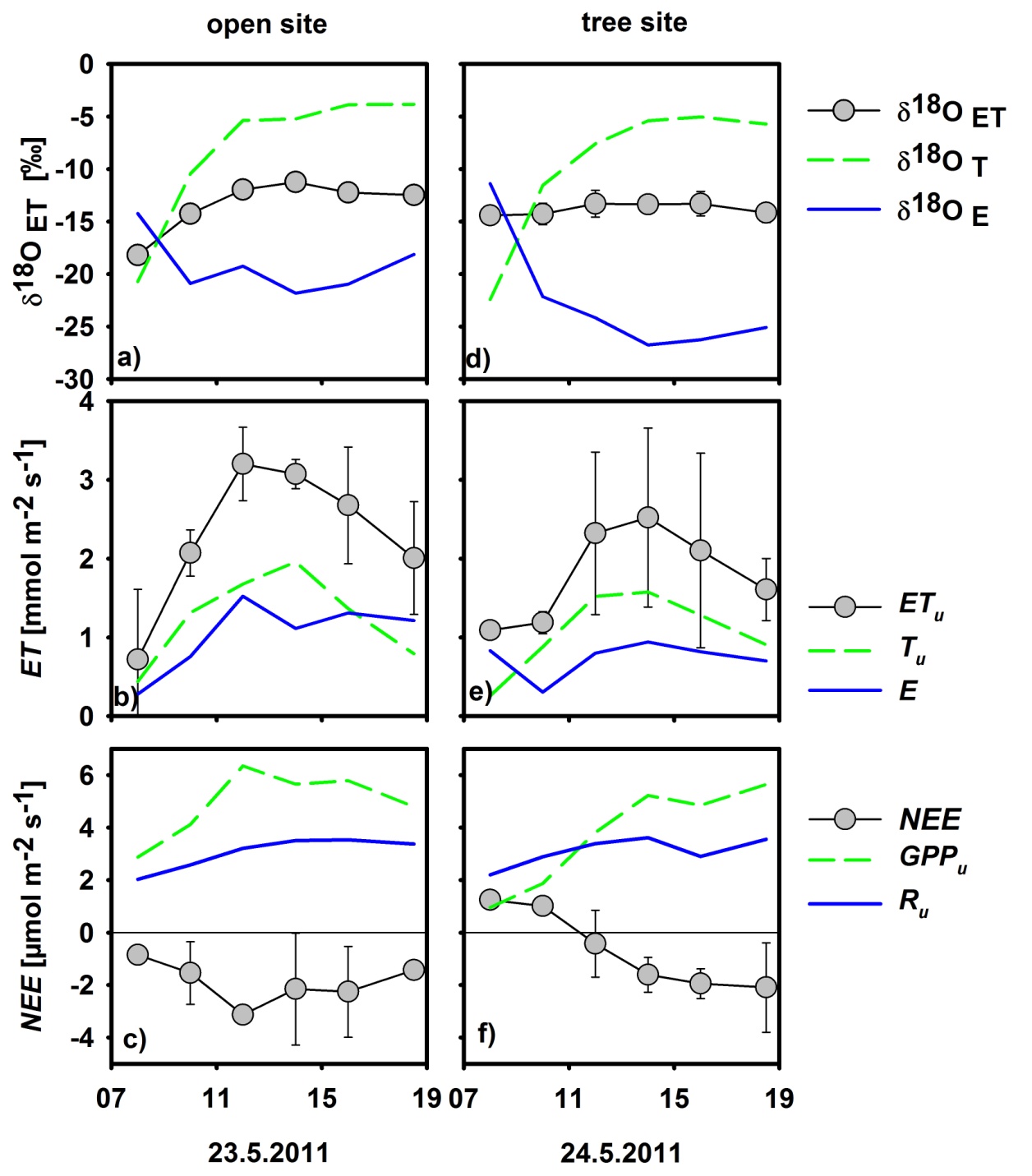


**Figure S1**: Typical diurnal courses of understory *δ^18^O_ET_*, *ET*, *NEE* and its components *E*, *T* , *δ^18^O_E_*, *δ^18^O_T_*, and *GPP*, *R_eco_* on 23. and 24. May 2011. Left column at the open site, right column at the tree site. a, d) Oxygen isotope signatures of measured *ET* (grey circles, n=3 ± SE) and modeled *E* and *T* (blue line and green dashed line). b, e) Fluxes of measured *ET* (grey circles, n=3 ± SD) and modeled *E* and *T* (blue line and green dashed line). c, f) Fluxes of measured net understory CO_2_ exchange (*NEE_u_*; grey circles, n=3 ± SD) and understory respiration (*R_u_*; blue circles, n=3 ± SD) and estimated understory plant CO_2_ uptake (*GPP_u_*; green dashed line).

**Table S1:** Oxygen isotope signatures of soil water [‰] in 0.5, 2, 5, 10, 15, 20 and 40 cm depth on bare soil and vegetation plots at the open and tree site between 7.4. and 21.11.2011.

| Sampling date | Depth (cm) | Open site | | | | Tree site | | | |
| --- | --- | --- | --- | --- | --- | --- | --- | --- | --- |
|  |  | Bare soil  (MW±SE) | | Vegetation (MW±SE) | | Bare soil (MW±SE) | | Vegetation (MW±SE) | |
| 7./8.4.2011 | 0.5 | -1.9 | 2.3 | -1.5 | 2.5 | 1.1 | 1.2 | -4.2 | 0.8 |
|  | 2 | -3.8 | 2.7 | -3.3 | 1.1 | -5.6 | 0.1 | -5.9 | 0.1 |
|  | 5 | -3.5 | 1.3 | -4.5 | 0.4 | -5.8 | 0.1 | -6.4 | 0.1 |
|  | 10 | -5.2 | 0.4 | -5.3 | 0.7 | -5.6 | 0.6 | -6.5 | 0.1 |
|  | 15 | -5.4 | 0.0 | -5.5 | 0.8 | -5.8 | 0.1 | -7.1 | 0.7 |
|  | 20 | -3.0 | 3.6 | -5.3 | 1.0 | -5.7 | 0.4 | -6.9 | 0.5 |
|  | 40 | -5.5 | 0.4 | -5.6 | 0.6 | -5.6 | 0.4 | -7.6 | 0.2 |
| 12/13.4.2011 | 0.5 | -2.5 | 1.2 | -2.8 | 0.4 | 3.5 | 0.8 | -0.2 | 0.4 |
|  | 2 | 3.3 | 1.3 | -2.5 | 0.4 | 3.6 | 0.1 | -0.1 | 0.2 |
|  | 5 | -0.3 | 0.4 | -5.4 | 1.3 | -1.7 | 1.6 | -3.3 | 2.2 |
|  | 10 | -6.0 | 0.1 | -6.8 | 0.4 | -5.6 | 0.0 | -6.7 | 0.0 |
|  | 15 | -5.8 | 0.4 | -6.7 | 0.1 | -5.9 | 0.1 | -6.6 | 0.5 |
|  | 20 | -6.1 | 0.1 | -7.0 | 0.3 | -6.0 | 0.1 | -7.0 | 0.2 |
|  | 40 | -6.2 | 0.1 | -6.5 | 0.0 | -6.4 | 0.3 | -7.0 | 0.2 |
| 18.4.2011 | 0.5 | 0.2 | 1.3 | 0.4 | 0.0 | n.a. | n.a. | n.a. | n.a. |
|  | 2 | 5.4 | 0.3 | 0.1 | 0.8 | n.a. | n.a. | n.a. | n.a. |
|  | 5 | n.a. | n.a. | -0.8 | 0.6 | n.a. | n.a. | n.a. | n.a. |
|  | 10 | -3.0 | 0.2 | -4.3 | 1.5 | n.a. | n.a. | n.a. | n.a. |
|  | 15 | -5.1 | 0.0 | -5.2 | 0.4 | n.a. | n.a. | n.a. | n.a. |
|  | 20 | -5.7 | 0.2 | -5.6 | 0.0 | n.a. | n.a. | n.a. | n.a. |
|  | 40 | -6.1 | 0.4 | -6.8 | 0.3 | n.a. | n.a. | n.a. | n.a. |
| 25.4.2011 | 0.5 | 2.2 | 0.6 | -0.7 | 0.8 | n.a. | n.a. | n.a. | n.a. |
|  | 2 | -3.5 | 2.5 | -3.1 | 0.1 | n.a. | n.a. | n.a. | n.a. |
|  | 5 | -3.9 | 0.8 | -5.4 | 0.8 | n.a. | n.a. | n.a. | n.a. |
|  | 10 | -3.7 | 0.5 | -5.5 | 0.2 | n.a. | n.a. | n.a. | n.a. |
|  | 15 | -4.4 | 0.1 | -5.1 | 0.1 | n.a. | n.a. | n.a. | n.a. |
|  | 20 | -3.8 | 1.8 | -5.6 | 0.3 | n.a. | n.a. | n.a. | n.a. |
|  | 40 | -6.1 | 0.0 | -5.9 | 0.0 | n.a. | n.a. | n.a. | n.a. |
| 2./3.5.2011 | 0.5 | 4.4 | 0.8 | -4.2 | 0.4 | -1.7 | 0.2 | -4.7 | 0.0 |
|  | 2 | -1.9 | 0.4 | -4.2 | 0.5 | -4.0 | 0.3 | -5.7 | 0.3 |
|  | 5 | -4.6 | 0.3 | -6.2 | 0.1 | -5.4 | 0.3 | -6.1 | 0.4 |
|  | 10 | -5.4 | 0.7 | -7.8 | 0.5 | -5.0 | 1.2 | -5.3 | 0.1 |
|  | 15 | n.a. | n.a. | -7.7 | 0.1 | -4.4 | 0.0 | -5.2 | 0.5 |
|  | 20 | -5.2 | 0.4 | -8.0 | 0.5 | -4.8 | 0.0 | -4.9 | 0.4 |
|  | 40 | -5.5 | 0.1 | -6.4 | 0.4 | -5.0 | 0.1 | -5.7 | 0.1 |
| 23./24.5.2011 | 0.5 | 1.9 | 1.5 | 1.8 | 2.1 | 1.8 | 0.3 | -1.0 | 1.2 |
|  | 2 | -3.9 | 1.4 | -1.7 | 0.1 | 0.0 | 1.8 | -1.1 | 1.0 |
|  | 5 | -2.1 | 1.8 | -3.2 | 0.5 | -1.1 | 0.6 | -3.7 | 0.9 |
|  | 10 | -4.6 | 2.5 | -5.3 | 0.8 | -4.6 | 1.7 | -3.9 | 1.1 |
|  | 15 | -3.0 | 0.5 | -4.1 | 0.1 | -5.4 | 1.5 | -4.1 | 0.1 |
|  | 20 | -4.1 | 1.5 | -4.9 | 0.2 | -3.3 | 0.9 | -3.0 | 0.1 |
|  | 40 | -5.6 | 1.8 | -3.9 | 1.2 | -4.4 | 0.2 | -4.6 | 0.2 |
| 25./27.5.2011 | 0.5 | 1.2 | 0.6 | 0.1 | 0.2 | 0.8 | 1.2 | -0.5 | 1.9 |
|  | 2 | -4.8 | 4.7 | -1.8 | 1.9 | -1.3 | 0.2 | -3.2 | 2.4 |
|  | 5 | -5.6 | 2.2 | -2.0 | 1.0 | -3.3 | 1.7 | -3.8 | 1.1 |
|  | 10 | -7.5 | 2.1 | -2.8 | 0.5 | -4.9 | 0.1 | -5.9 | 1.3 |
|  | 15 | -5.2 | 0.7 | -3.9 | 0.8 | -6.6 | 1.3 | -5.6 | 1.5 |
|  | 20 | -4.4 | 0.6 | -3.9 | 0.1 | -4.7 | 2.1 | -5.2 | 1.2 |
|  | 40 | -4.9 | 1.7 | -4.2 | 0.7 | -5.3 | 0.4 | -5.8 | 0.8 |
| 31.5.2011 | 0.5 | 4.7 | 0.8 | 2.4 | 1.2 | n.a. | n.a. | n.a. | n.a. |
|  | 2 | -2.3 | 3.4 | -0.1 | 0.7 | n.a. | n.a. | n.a. | n.a. |
|  | 5 | -3.5 | 1.6 | -7.6 | 4.6 | n.a. | n.a. | n.a. | n.a. |
|  | 10 | -4.6 | 0.3 | -5.6 | 3.1 | n.a. | n.a. | n.a. | n.a. |
|  | 15 | -4.5 | 1.1 | -4.1 | 1.5 | n.a. | n.a. | n.a. | n.a. |
|  | 20 | -4.6 | 2.1 | -7.4 | 3.1 | n.a. | n.a. | n.a. | n.a. |
|  | 40 | -4.7 | 1.6 | -5.7 | 0.3 | n.a. | n.a. | n.a. | n.a. |
| 1./2.6.2011 | 0.5 | -0.3 | 4.6 | -0.6 | 6.6 | -1.4 | 2.9 | -0.2 | 0.8 |
|  | 2 | -3.1 | 2.5 | -1.9 | 0.7 | -3.2 | 1.7 | -1.7 | 3.5 |
|  | 5 | -4.2 | 1.6 | -4.0 | 3.2 | -1.1 | 0.9 | -4.1 | 0.1 |
|  | 10 | -4.6 | 1.1 | -5.6 | 1.1 | -4.4 | 1.8 | -4.9 | 0.5 |
|  | 15 | -5.5 | 2.0 | -2.5 | 0.2 | -3.8 | 2.1 | -5.1 | 2.2 |
|  | 20 | -4.0 | 1.8 | -3.6 | 1.2 | -3.9 | 2.2 | -6.0 | 0.5 |
|  | 40 | -3.7 | 0.6 | -3.0 | 0.4 | -3.7 | 3.1 | -3.4 | 2.0 |
| 9./11.6.2011 | 0.5 | n.a. | n.a. | 4.9 | 1.3 | n.a. | n.a. | 4.1 | 0.5 |
|  | 2 | 7.4 | 1.2 | -1.8 | 0.6 | 8.0 | 2.0 | -1.2 | 2.0 |
|  | 5 | -0.6 | 0.7 | -4.6 | 1.4 | 1.4 | 1.5 | -2.7 | 1.0 |
|  | 10 | -2.1 | 0.9 | -4.1 | 0.4 | -5.2 | 4.1 | -5.9 | 1.2 |
|  | 15 | -1.2 | 1.5 | -6.2 | 0.9 | -2.6 | 0.3 | -3.9 | 1.3 |
|  | 20 | -1.9 | 0.5 | -5.0 | 2.0 | -2.7 | 3.0 | -3.9 | 1.0 |
|  | 40 | -4.6 | 0.8 | -5.5 | 0.2 | -3.7 | 0.5 | -4.5 | 1.9 |
| 14.6.2011 | 0.5 | n.a. | n.a. | 7.0 | 0.8 | -2.2 | 1.5 | 3.2 | 1.2 |
|  | 2 | -2.2 | 1.0 | 7.3 | 1.3 | 5.2 | 1.4 | 6.5 | 1.2 |
|  | 5 | 5.8 | 0.9 | 1.6 | 0.5 | 5.1 | 0.8 | -6.5 | 1.0 |
|  | 10 | -1.5 | 2.1 | -1.5 | 0.3 | -3.1 | 4.5 | -1.5 | 0.1 |
|  | 15 | -1.6 | 0.1 | -1.4 | 0.1 | -2.6 | 3.4 | -4.8 | 1.3 |
|  | 20 | -5.0 | 3.1 | -1.6 | 1.3 | -3.2 | 0.8 | -4.2 | 3.0 |
|  | 40 | -2.3 | 1.2 | -6.7 | 2.5 | -0.8 | 0.8 | -2.6 | 2.9 |
| 14.9.2011 | 0.5 | n.a. | n.a. | n.a. | n.a. | n.a. | n.a. | -0.6 | 2.6 |
|  | 2 | n.a. | n.a. | n.a. | n.a. | n.a. | n.a. | 5.4 | 1.5 |
|  | 5 | n.a. | n.a. | 5.1 | 1.9 | n.a. | n.a. | 5.6 | 1.2 |
|  | 10 | n.a. | n.a. | 2.6 | 2.0 | n.a. | n.a. | -0.5 | 1.6 |
|  | 15 | n.a. | n.a. | -0.2 | 0.3 | n.a. | n.a. | -1.2 | 0.8 |
|  | 20 | n.a. | n.a. | -2.9 | 0.4 | n.a. | n.a. | 1.5 | 1.0 |
|  | 40 | n.a. | n.a. | -1.9 | 0.8 | n.a. | n.a. | -0.6 | 2.6 |
| 28./29.10.2011 | 0.5 | -0.7 | 2.2 | -1.6 | 0.4 | 1.6 | 1.7 | -2.1 | 0.3 |
|  | 2 | -3.9 | 0.2 | -2.8 | 2.0 | -1.7 | 0.2 | -2.9 | 0.3 |
|  | 5 | -4.0 | 0.2 | -2.9 | 0.5 | -2.5 | 0.7 | -4.5 | 0.3 |
|  | 10 | -5.3 | 0.7 | -5.0 | 0.5 | -3.5 | 2.9 | -5.6 | 1.7 |
|  | 15 | -4.2 | 0.4 | -2.7 | 0.3 | -2.0 | 0.4 | -2.7 | 1.2 |
|  | 20 | -5.7 | 0.2 | -4.2 | 0.5 | -5.1 | 0.3 | -2.1 | 1.6 |
|  | 40 | -4.4 | 0.8 | -6.1 | 1.3 | -4.2 | 0.3 | -2.9 | 0.2 |
| 4./5.11.2011 | 0.5 | n.a. | n.a. | -6.9 | 0.8 | n.a. | n.a. | -6.9 | 1.8 |
|  | 2 | n.a. | n.a. | -5.8 | 1.5 | n.a. | n.a. | -4.5 | 2.0 |
|  | 5 | n.a. | n.a. | -4.2 | 0.1 | n.a. | n.a. | -3.4 | 1.3 |
|  | 10 | n.a. | n.a. | -3.7 | 0.8 | n.a. | n.a. | -4.4 | 1.0 |
|  | 15 | n.a. | n.a. | -3.8 | 1.5 | n.a. | n.a. | -7.2 | 1.3 |
|  | 20 | n.a. | n.a. | -4.1 | 0.3 | n.a. | n.a. | -4.7 | 0.2 |
|  | 40 | n.a. | n.a. | -5.7 | 0.4 | n.a. | n.a. | -5.1 | 0.6 |
| 6./7.11.2011 | 0.5 | -1.0 | 0.9 | -4.9 | 0.9 | 0.5 | 1.9 | -5.5 | 1.7 |
|  | 2 | -3.8 | 1.9 | -4.8 | 0.7 | -2.8 | 0.2 | -4.2 | 0.3 |
|  | 5 | -4.1 | 1.8 | -3.4 | 0.9 | -3.2 | 0.4 | -5.4 | 0.4 |
|  | 10 | -3.1 | 1.5 | -3.5 | 1.7 | -4.4 | 1.8 | n.a. | n.a. |
|  | 15 | -3.7 | 1.2 | -3.1 | 3.0 | -3.1 | 0.4 | -3.3 | 0.6 |
|  | 20 | -3.7 | 0.5 | -3.3 | 1.3 | -5.4 | 0.3 | -3.1 | 1.0 |
|  | 40 | -3.7 | 0.8 | -3.2 | 0.7 | -4.3 | 0.6 | -3.3 | 0.6 |
| 16./17.11.2011 | 0.5 | -0.4 | 0.5 | -3.7 | 0.9 | -1.6 | 1.3 | -4.5 | 0.8 |
|  | 2 | -5.9 | 1.1 | -4.9 | 0.9 | -1.7 | 2.6 | -4.6 | 0.5 |
|  | 5 | -4.9 | 2.1 | -2.9 | 1.3 | -3.2 | 1.5 | -3.9 | 0.5 |
|  | 10 | -3.5 |  | -3.0 | 1.1 | -2.7 | 1.5 | -4.0 | 2.3 |
|  | 15 | -4.4 | 1.9 | -2.6 | 5.5 | -3.0 | 0.6 | -3.6 | 0.5 |
|  | 20 | -3.4 | 0.4 | -3.2 | 1.4 | -3.9 | 0.5 | -3.3 | 1.1 |
|  | 40 | -3.7 | 0.6 | -3.9 | 0.4 | -3.6 | 0.9 | -2.4 | 0.9 |
| 20./21.11.2011 | 0.5 | n.a. | n.a. | -4.8 | 1.4 | n.a. | n.a. | -5.7 | 1.2 |
|  | 2 | n.a. | n.a. | -3.3 | 0.2 | n.a. | n.a. | -5.1 | 0.4 |
|  | 5 | n.a. | n.a. | -5.1 | 0.6 | n.a. | n.a. | -5.6 | 0.3 |
|  | 10 | n.a. | n.a. | n.a. | n.a. | n.a. | n.a. | n.a. | n.a. |
|  | 15 | n.a. | n.a. | -3.7 | 0.5 | n.a. | n.a. | -2.8 | 0.6 |
|  | 20 | n.a. | n.a. | -2.9 | 1.0 | n.a. | n.a. | -3.2 | 1.0 |
|  | 40 | n.a. | n.a. | -2.9 | 0.8 | n.a. | n.a. | -3.6 | 0.3 |
